# Randomized Field Trial to Assess the Safety and Efficacy of Dihydroartemisinin-Piperaquine for Seasonal Malaria Chemoprevention in School-Aged Children in Bandiagara, Mali

**DOI:** 10.1093/infdis/jiad387

**Published:** 2023-09-08

**Authors:** Karim Traore, Drissa Coulibaly, Abdoulaye K Kone, Boureima Guindo, Souleymane Traore, Kindie Kouriba, Moussa Djimde, Mahamadou Ali Thera

**Affiliations:** Malaria Research and Training Center, University of Sciences, Techniques, and Technologies, Bamako, Mali; Malaria Research and Training Center, University of Sciences, Techniques, and Technologies, Bamako, Mali; Malaria Research and Training Center, University of Sciences, Techniques, and Technologies, Bamako, Mali; Malaria Research and Training Center, University of Sciences, Techniques, and Technologies, Bamako, Mali; Malaria Research and Training Center, University of Sciences, Techniques, and Technologies, Bamako, Mali; Malaria Research and Training Center, University of Sciences, Techniques, and Technologies, Bamako, Mali; Malaria Research and Training Center, University of Sciences, Techniques, and Technologies, Bamako, Mali; Malaria Research and Training Center, University of Sciences, Techniques, and Technologies, Bamako, Mali

**Keywords:** DHA-PQ, malaria, Mali, school-aged children, SMC

## Abstract

**Background:**

Owing to the increased cases of malaria in older children, the World Health Organization has recently recommended extending seasonal malaria chemoprevention (SMC) to children >5 years of age and using other effective drugs for malaria. In this study, we report the safety and efficacy of dihydroartemisinin-piperaquine (DHA-PQ) for SMC in school-aged children in Mali.

**Method:**

This randomized, controlled trial included 345 participants aged 6–15 years randomized to receive DHA-PQ, sulfadoxine-pyrimethamine plus amodiaquine (SP-AQ), or no chemoprevention (albendazole) at a 1:1:1 ratio. Four rounds of SMC were conducted from September to December 2021. The participants were assessed 7 days after each round for safety and efficacy of the interventions.

**Results:**

Abdominal pain (11.8% vs 29.2%), headache (11.2% vs 19.2%), and vomiting (5.7% vs 15.2%) were frequently reported in the DHA-PQ and SP-AQ arms. On Day 120 of follow up, the incidence of clinical malaria was 0.01 episodes/person-month in the DHA-PQ and SP-AQ arms and 0.17 episodes/person-month in the control arm (*P* < .0001). Gametocytes were detected in 37 participants in all arms.

**Conclusions:**

Children in DHA-PQ arm reported less adverse events compared to the SP-AQ arm. Both drugs were effective against clinical malaria and infection.

## Teaser

Extending the seasonal malaria chemoprevention (SMC) strategy to school-aged children and using alternative drugs are part of a recent recommendation by the WHO. We demonstrated the safety and efficacy of dihydroartemisinin-piperaquine for SMC in children aged 6–15 years.

Seasonal malaria chemoprevention (SMC) using sulfadoxine-pyrimethamine plus amodiaquine (SP-AQ) is implemented in areas where 60% malaria transmission occurs during the rainy season over 4 months. According to the 2012 recommendation of the World Health Organization (WHO), SMC targets children <5 years of age [1–3]. The large-scale implementation of SMC is a part of the national malaria control policies of several countries in West Africa. In several countries, school-aged children are currently not targeted by SMC. Some countries have recently extended it to children up to 10 years old, but it remains limited to some health districts and is not yet considered a nationwide policy. The potential age shift in malaria burden is supported by data published since 2014, indicating that the scale-up of interventions for malaria with significant impact on disease burden in children <5 years of age may lead to a shift in the population at risk for malaria [4, 5]. In Bandiagara, Mali, malaria burden is high among children ≤15 years of age [6]. Evidence from field trials and meta-analysis suggest that extending SMC to older children helps reduce the malaria burden, supporting the effectiveness of malaria elimination programs [7].

The 2022 WHO recommendations states that the target age can be defined as per the specific policy of each country according to the local epidemiology of the disease. Apart from the first-line medication for clinical malaria, other artemisinin-based combination therapies can also be used for SMC [8]. Dihydroartemisinin-piperaquine (DHA-PQ) is a long-acting, artemisinin-based, combination therapy recommended by the WHO for the treatment of uncomplicated malaria. It is an alternative drug that could be potentially used for SMC. Comparative studies of drugs used for SMC and the treatment of uncomplicated clinical malaria conducted in Senegal, The Gambia, Burkina Faso, Mali, and Uganda have demonstrated the efficacy of DHA-PQ and SP-AQ [9–13]. The rapid parasite clearance and gametocidal action of artemisinin may augment the value of DHA-PQ for SMC. This noninferiority, randomized, controlled trial assessed the safety and efficacy of DHA-PQ compared with SP-AQ for SMC targeting school-aged children in Bandiagara, Mali.

## METHODS

### Study Site, Period, and Type

We conducted a Phase IV, noninferiority, randomized trial that included children aged 6–15 years in Bandiagara, Mali, from September 2021 to September 2022. The participants were categorized into the following treatment arms: DHA-PQ, SP-AQ, and control (albendazole). Bandiagara is an area with a high incidence of malaria. It is approximately 400 miles northeast from its capital city, Bamako. Furthermore, it has 15 000 residents who survive largely on agriculture. Bandiagara has a mean annual rainfall of 600 mm mainly from June to November. Malaria is a mesohyperendemic disease with peak transmission season from September to November. The Malaria Research and Training Center (MRTC) has set up a clinical research center in 2002 in Bandiagara for the implementation of drug and vaccine trials. The National Malaria Control Program (NMCP) of Mali has been implementing SMC among children <5 years of age in Bandiagara since 2016. (The trial was registered in the Pan African Clinical Trials Registry, trial number PACTR202007754558749.)

### Study Population

The study population comprised children aged 6–15 years living in Bandiagara and enrolled in a basic school. The inclusion criteria were as follows: (1) children aged 6 to 15 enrolled in the Bandiagara Basic School; (2) available and able to make follow-up visits; (3) do not show any general signs of danger or other signs of severe and complicated *P*lasmodium *falciparum* malaria according to the WHO criteria; (4) informed consent of the parents or guardian of the child and children assent (13–15 years old) obtained; (5) no abnormalities in electrocardiogram ([EKG] QTc interval <450); (6) no known history of hypersensitivity reaction to the drugs used.

### Recruitment and Retention of Participants

The recruitment started with the community permission approach commonly used by MRTC [14]. School and local authorities, parents of children, and community leaders were informed about the study procedures and objectives. A meeting with the authorities was held to discuss all relevant information regarding the trial. Individual informed consent from the parents was obtained before inviting the volunteers (aged 6–15 years) to undergo the screening procedures at the clinical center. Relevant information about the study procedures was disclosed to the parents to reduce the risks for noncompliance and loss to follow-up. In addition, children's assent was obtained for those aged between 13 and 15 years. Clinical and biological assessments as well as EKG were performed for all consenting participants. The eligibility criteria were verified, and a list of screened and eligible children was generated.

### Enrollment and Randomization

Eligible children aged 6–15 years were enrolled and randomized to receive DHA-PQ, SP-AQ, or no chemoprevention (albendazole) at a 1:1:1 ratio. Computer-generated sequential codes were randomly and uniquely linked to each treatment arm. The codes were assigned to children in the order they were enrolled in the study. We adopted the double-blind design for this trial. The study drugs were administered by dedicated pharmacists not involved in further outcome assessment of the trial. There was a slight difference in the appearance of the study drugs. Drug administration was supervised by the investigator to reduce the risks of drug identification by the children and to prevent them from bringing the drugs home and discussing with others. Except for the pharmacists, the other investigators remained blinded to the treatment. However, because there was physical difference with the drugs and some children were able to identify the drug they are taking, the study conduct was single blind rather than full double blind.

### Interventions

The children received the interventions each month for 4 consecutive months. Each SMC round lasted for 3 days. In the DHA-PQ arm, DHA was administered at 4 mg/kg of body weight/day for 3 consecutive days and PQ was administered at 18 mg/kg of body weight/day for 3 consecutive days. In the SP-AQ arm, SP was administered on the first day at 25 mg/kg of body weight of sulfadoxine and 1.25 mg/kg of body weight of pyrimethamine, whereas AQ was administered at 10 mg/kg of body weight for 3 consecutive days.

Control arm participants received no chemoprevention. All participants in each group received albendazole at a dose of 400 mg/day monthly for 3 consecutive days/month to reduce any bias due to its impact on the trial endpoints.

In Mali, albendazole is used for the mass treatment of soil-transmitted helminth infection in children. Clinical studies have demonstrated that it has no effect on the clinical epidemiology of malaria. The use of albendazole as a control drug would potentially benefit our study population with a high prevalence of soil-transmitted helminth infection.

The drugs were administered under the direct supervision of pharmacists, and the participants were observed for 1 hour after the drug intake and then seen at the clinic after 7 days. The teachers and parents were encouraged to inform the study team of any ill participant for clinical evaluation and malaria diagnosis. Furthermore, cross-sectional surveys were conducted at baseline and then at 6 and 12 months after the first dose of SMC. Any clinical episodes of malaria were managed as per the Malian NMCP guidelines. We also encouraged the participants to adopt the preventive measures recommended by the aforementioned guidelines.

Sulfadoxine-pyrimethamine plus AQ (SP 500/25 mg and AQ 153 mg) was provided by the NMCP, and DHA-PQ (Eurartesim 320/40 mg [Alfasigma], prequalified by the WHO) and albendazole were obtained from a public pharmacy. Sulfadoxine-pyrimethamine plus AQ was manufactured by Guilin Pharma, which is the principal manufacturer of the SP-AQ used by NMCP in SMC in Mali since 2020.

### Outcome Assessments

#### Safety Primary Outcome

Solicited (fever, malaise, headache, nausea, vomiting, abdominal pain, diarrhea, anorexia, myalgia, and dizziness) and unsolicited symptoms were assessed 7 days after each SMC drug administration. The adverse events (AEs) were evaluated and classified according to their intensity (mild, moderate, or severe) and their relationship to the study products (related or not related).

The EKG was performed at screening to exclude children with any preexisting anomalies and after SMC round 4 to detect any QTc interval prolongation. The serum titers of alanine aminotransferase (ALT) were measured on Day 7 after the start of SMC round 3 to assess the hepatotoxicity of the investigational products.

#### Efficacy Primary Outcome

During the 4 SMC rounds and 30 days after SMC round 4 (Day 120), the incidence of clinical malaria was measured. Clinical malaria was defined as the presence of asexual forms of *P falciparum* in a thick smear and associated with symptoms such as fever/chills, myalgia/asthenia, headache, nausea, vomiting, abdominal pain, and diarrhea without any signs of danger or severe illness. The prevalence of malaria infection and gametocyte carriage were determined monthly on Day 1 of each SMC round and at the cross-sectional surveys conducted on months 6 and 12 by preparing thick smears.

### Laboratory Procedures

At enrollment, thick and thin smears as well as dried blood spots were collected using 3MM Whatman filter papers from each participant via venipuncture before drug administration. Malaria thick smears were Giemsa stained, and parasites were counted against 300 leukocytes to provide parasite counts/mm^3^, assuming a leukocyte count of 7500/mm^3^. The participants’ hemoglobin level was determined at baseline, on Day 7 after the start of each SMC round, and at cross-sectional surveys using a hemoglobin analyzer (HemoCue Inc., Cypress, CA). The same tests were repeated in case of a clinical malaria episode. Furthermore, a β-human chorionic gonadotropin pregnancy test was conducted in female participants >13 years of age on day 1 of each SMC round before drug administration. A 5-mL venous blood sample was collected from each participant to assess hepatotoxicity by measuring the serum titer of ALT on Day 7 after the start of SMC round 3.

### Statistical Methods

The sample size was determined based on a recent SMC study involving school-aged children in Mali using artesunate plus AQ, which showed a 20% incidence of uncomplicated clinical malaria in the control arm [15]. Using an inverse sine transform approximation and assuming α risk error of 5% and a power of 83%, 70% reduction in the incidence of uncomplicated malaria in the SMC arms compared with the control arm, and overall rate of loss to follow up and noncompliance with the study procedures of 10%, it was estimated that a sample size of 345 subjects for the 3 arms (115 subjects per arm) was required.

For efficacy analysis, the incidence of clinical malaria and proportion of malaria infection episodes between the study arms were compared using χ^2^ test and relative risk ratios. All tests were 2-sided, and the incidence of clinical malaria was determined using the number of episodes by person-time after 12 months of follow up.

### Ethical and Regulatory Considerations

The trial was conducted in accordance with the ICH-GCP guidelines and local Malian laws (law No. 09-059/AN-RM on biomedical research and law No. 2013-015/AN-RM for personal data protection). The ethics committee of the Faculty of Medicine, Odonto-Stomatology, and Pharmacy, Mali, as well as the Ministry of Health, Mali, approved this clinical trial. A local ethical advisor was appointed by the sponsor to assist the participants. All participants provided informed consent before enrollment. Periodic safety reports were provided to the Data Safety Monitoring Board (DSMB) before the subsequent SMC round. The trial safety oversight was performed by the DSMB and independent monitor.

## RESULTS

**Figure 1.**
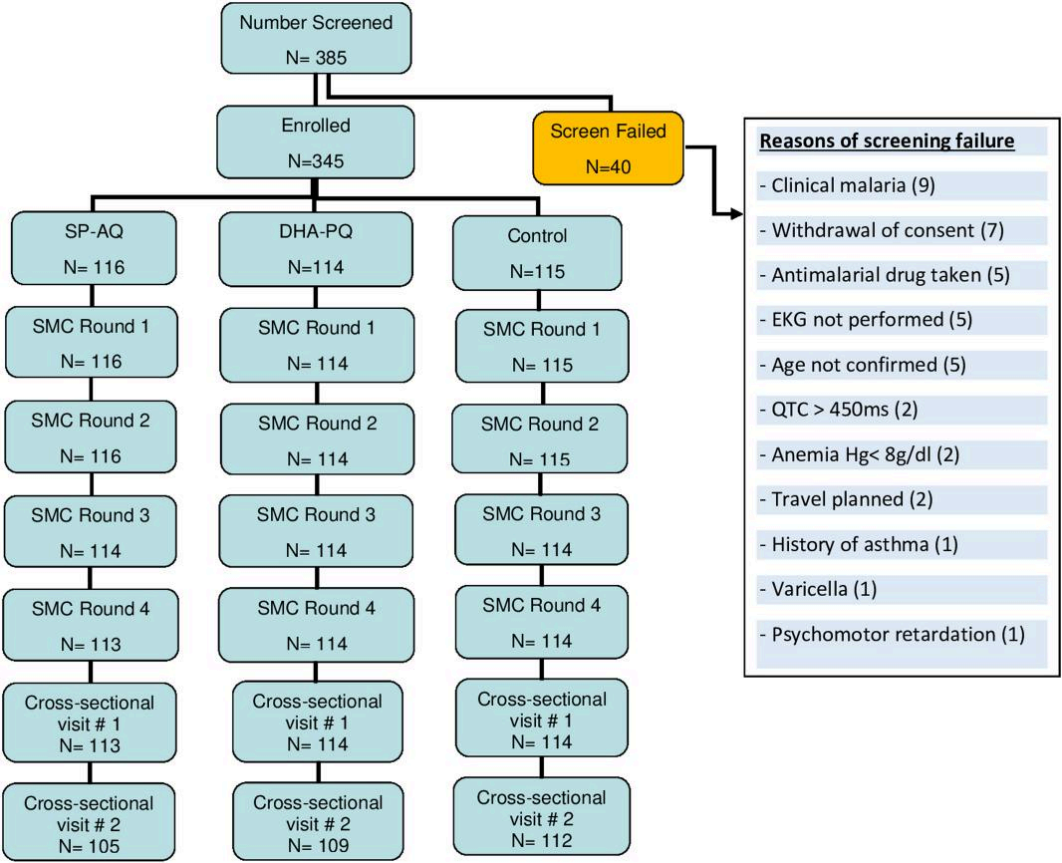
Study profile showing the overview of the study screening and follow up. DHA-PQ, dihydroartemisinin-piperaquine; SMC, seasonal malaria chemoprevention; SP-AQ, sulfadoxine-pyrimethamine plus amodiaquine.

From September 12 to 23, 2020, a total of 385 volunteers were screened, 345 (185 females and 160 males) of whom were enrolled in the study and allocated to the following arms: SP-AQ (n = 115), DHA-PQ (n = 115), or control (n = 115). At SMC round 1, one participant in the DHA-PQ arm mistakenly received SP-AQ owing to pharmacy error and remained definitively in the SP-AQ arm. All the participants successfully completed SMC rounds 1 and 2. The total rate of loss to follow up was 1.15% at SMC round 4% and 5.5% at the end of follow up (Figure 1). The sex ratio was 1.15 in favor of the female participants, and the median age was 10 years in the SP-AQ and DHA-PQ arms and 9 years in the control arm (Table 1).

**Table 1.** Demographic and Baseline Characteristics of Participants

Characteristics		SP-AQ	DHA-PQ	Control (Albemdazol)	*P*
		Mean ± SD	Mean ± SD	Mean ± SD	
Age (years)		10 ± 2.5	10.1 ± 2.4	9.7 ± 2.7	.559
Temperature (^o^C)		36.5 ± 0.4	36.5 ± 0.4	36.5 ± 0.4	.788
Systolic blood pressure (mmHg)		105.3 ± 12.2	104 ± 13.6	102.2 ± 12	.931
Diastolic blood pressure (mmHg)		74.5 ± 8.5	71.5 ± 9.7	70.7 ± 9.3	.158
Pulse (bpm)		89.8 ± 11.8	89.9 ± 12.6	90.9 ± 11.5	.163
Respiratory rates		25.8 ± 3.7	26.1 ± 4.1	26 ± 4.0	.709
QTC interval		398 ± 19.4	395.7 ± 17	396.7 ± 18.3	.863
Weight (kg)		31.9 ± 11.1	31.5 ± 9.8	30.5 ± 10.8	.565
Sex F/M: ratio		66/50 (1.3)	54/60 (0.9)	65/50 (1.3)	.2616
Use of bed net	Yes: n (%)	113 (99.1)	112 (96.6)	113 (98.3)	.370
	No: n (%)	1 (0.9)	4 (3.4)	2 (1.7)	

Abbreviations: DHA-PQ, dihydroartemisinin-piperaquine; SD, standard deviation; SP-AQ, sulfadoxine-pyrimethamine plus amodiaquine.

Abdominal pain (134 vs 54 vs 35), headache (87 vs 51 vs 27), vomiting (70 vs 26 vs 6), and nausea (10 vs 6 vs 0) were the symptoms frequently reported by the participants in the SP-AQ, DHA-PQ, and control arms. In addition, fever, anorexia, dizziness, myalgia, and diarrhea were reported at low frequencies. The cumulative numbers of solicited symptoms are summarized in Table 2. All solicited events were mild to moderate severity (Supplementary Material 1), and the majority was assessed as study drug related (Supplementary Material 2).

**Table 2.** Number and Percentage of Participants Experiencing Solicited Events by Symptom, Round, Treatment Arm, Intention to Treat

SMC Rounds	SMC Round 1			SMC Round 2			SMC Round 3			SMC Round 4		
Treatment Arms	SP-AQ n = 116	DHA-PQ n = 114	AL n = 115	SP-AQ n = 116	DHA-PQ n = 114	AL n = 115	SP-AQ n = 114	DHA-PQ n = 114	AL n 114	SP-AQ n = 113	DHA-PQ n = 114	AL n = 114
Symptom	n (%)	n (%)	n (%)	n (%)	n (%)	n (%)	n (%)	n (%)	n (%)	n (%)	n (%)	n (%)
Fever	0	3 (0.03)	1 (0.01)	1 (0.01)	1 (0.01)	0	0	1 (0.01)	0	0	1 (0.01)	1 (0.01)
Nausea	3 (0.02)	2 (0.02)	0	7 (0.06)	1 (0.01)	0	0	3 (0.03)	0	0	0	0
Vomiting	23 (0.2)	5 (0.04)	5 (0.04)	21 (0.2)	7 (0.06)	1 (0.01)	19 (0.2)	8 (0.07)	0	7 (0.07)	6 (0.05)	2 (0.02)
Headache	21 (0.2)	18 (0.2)	10 (0.1)	35 (0.3)	17 (0.15)	8 (0.07)	23 (0.2)	6 (0.05)	3 (0.03)	9 (0.08)	10 (0.09)	6 (0.05)
Abdominal pain	53 (0.4)	18 (0.2)	13 (0.1)	34 (0.3)	15 (0.1)	2 (0.02)	24 (0.2)	10 (0.1)	12 (0.1)	23 (0.2)	11 (0.10)	9 (0.08)
Dizziness	0	2 (0.02)	0	3 (0.02)	4 (0.03)	1 (0.01)	3 (0.03)	0	0	3 (0.03)	0	0
Myalgia	3 (0.02)	0	1 (0.01)	2 (0.02)	0	2 (0.02)	2 (0.02)	0	0	1 (0.01)	0	0
Diarrhea	1 (0.01)	0	1 (0.01)	0	0	0	0	3 (0.03)	0	0	0	0
Anorexia	3 (0.02)	0	0	0	1 (0.01)	0	1 (0.0)	1 (0.01)	0	0	1 (0.01)	0

Abbreviations: AL, Albendazole; DHA-PQ, dihydroartemisinin-piperaquine; SMC, seasonal malaria chemoprevention; SP-AQ, sulfadoxine-pyrimethamine plus amodiaquine.

**Figure 2.**
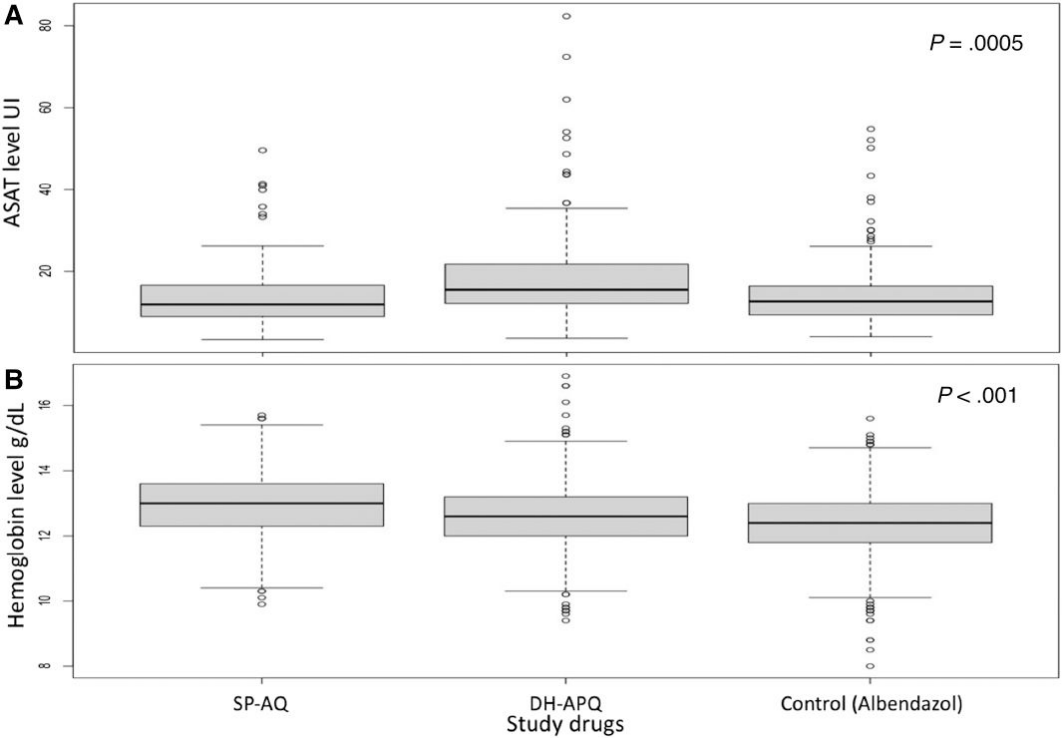
Besswarm plot comparing the level of alanine aminotransferase (ASAT) (*A*) at the third round of seasonal malaria chemoprevention (SMC) and the hemoglobin level (*B*) according to the study drug over the follow-up period. DHA-PQ, dihydroartemisinin-piperaquine; SP-AQ, sulfadoxine-pyrimethamine plus amodiaquine.

Comparison of the 3 arms showed that the median (Q1–Q3) ALT level was higher in the DHA-PQ arm (15.55 IU/L, 12.2–21.70) than in the SP-AQ (12.00 IU/L, 9.0–16.70) and control (12.70 IU/L, 9.4–16.45) arms (*P* = .0006) (Figure 2*A*). At baseline, the mean ± standard deviation of the hemoglobin level was similar among the arms (SP-AQ, 12.7 ± 0.9; DHA-PQ, 12.6 ± 1.0; and control, 12.5 ± 1.0). At Day 7 after SMC round 4, the mean hemoglobin level was lower in the control arm, with 1 person exhibiting a level of 7 g/dL (Figure 2*B*); the difference was statistically significant (*P* < .001).

**Figure 3.**
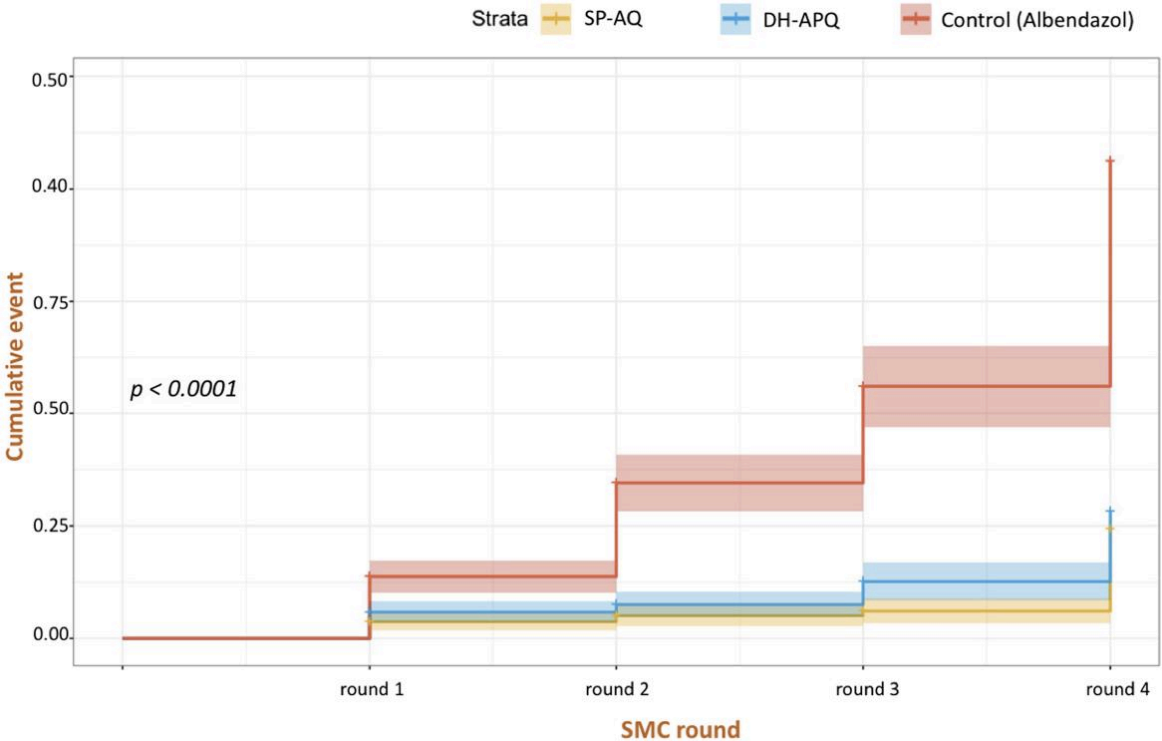
Cumulative incidences of malaria infection by treatment arm from the first round of SMC to after the fourth round of SMC.

The control arm had a higher incidence of clinical malaria regardless of the SMC round (Table 3). On Day 30 after SMC round 4, the incidence was 0.01 episodes/person-month in the SP-AQ and DHA-PQ arms and 0.17 episodes/person-month in the control arm. At 12 months of follow up, the incidence rates were 0.52, 0.46, and 1.12 episodes/person-year in the SP-AQ, DHA-PQ, and control arms, respectively. The differences were statistically significant between the DHA-PQ and control arms (*P* < .0001) and the SP-AQ and control arms (*P* < .001). The DHA-PQ and SP-AQ arms were similar in terms of clinical malaria incidences (*P* = .6) (Table 3). Parasitemia was more frequent in the control arm. In addition, the cumulative incidence of malarial infection was higher in this arm (Figure 3).

**Table 3.** Clinical Malaria Incidence per Treatment Arm at 4 and 12 months After the Start of the First SMC Round

Treatment Arms	SP-AQ	DHA-PQ	Control	Total	*P* Values		
					SP-AQ vs Control	DHA-PQ vs Control	SP-AQ vs DHA-PQ
Follow-up at 4 months							
Number of episodes, incidence/person-month	5 (0.01)	5 (0.01)	77 (0.17)	87 (0.06)	<.0001	<.0001	.7
Person-time, months	454	460	452	1366	…		
Follow-up at 12 months							
Number of episodes, incidence/person-year	59 (0.52)	53 (0.46)	130 (1.12)	242 (0.70)	<.0001	<.0001	.6
Person-time, years	114	115.5	115.7	345.2	…		

Abbreviations: DHA-PQ, dihydroartemisinin-piperaquine; SMC, seasonal malaria chemoprevention; SP-AQ, sulfadoxine-pyrimethamine plus amodiaquine.

Furthermore, the prevalence of gametocyte was higher in the control arm (n = 29, 78.4%) than in the SP-AQ (n = 3, 8.1%) and DHA-PQ (n = 5, 13.5%) arms (Table 4).

**Table 4.** Distribution of Gametocytes Prevalence by Treatment Group

Treatment Arm	Treatment Arm: SP-AQ		Treatment Arm: DHA-PQ		Treatment Arm: Control	
	n	%	n	%	n	%
From days 1–30 (during and after SMC round 1), *P* = .807						
Gametocyte^+^	2	22.2	3	33.3	4	44.4
From day 31–60 (during and after SMC round 2), *P* = .0004						
Gametocyte^+^	1	9.1	0	0.0	10	90.9
From day 61–90 (during and after SMC round 3), *P* = .0017						
Gametocyte^+^	0	0.0	1	11.1	8	88.9
From day 91–120 (during and after SMC round 4), *P* = .0072						
Gametocyte^+^	0	0.0	1	12.5	7	87.5
From day 1–120 (during and after All SMC rounds), *P* < .0001						
Gametocyte^+^	3	8.1	5	13.5	29	78.4

Abbreviations: DHA-PQ, dihydroartemisinin-piperaquine; SMC, seasonal malaria chemoprevention; SP-AQ, sulfadoxine-pyrimethamine plus amodiaquine.

## DISCUSSION

This noninferiority, blinded, randomized, controlled trial was conducted to assess the safety and efficacy of DHA-PQ for SMC targeting school-aged children. To maintain the blinded design of the trial, the study drugs were administered by dedicated pharmacists who were not involved in further assessments of the participants. The drugs were slightly different in terms of physical appearance, but supervised administration at the clinic was ensured to minimize the risks of drug identification by the participants. However, it was difficult to maintain all participants blinded about the treatment received. The 4 SMC rounds were successfully conducted, and the total rate of loss to follow up at the last round was 1.5%. This value is less than that reported in Burkina Faso by Zongo et al [10], which was approximately 7%–9%. This difference could be explained by the experience and capacities of the study team to recruit and maintain participants in the study.

Abdominal pain, headache, vomiting, myalgia, dizziness, nausea, and diarrhea were the solicited symptoms recorded by order of importance. The frequencies of these symptoms decreased from SMC round 1 to round 4 and were higher in the SP-AQ arm, followed by the DHA-PQ and control arms. Most of the cases of loss to follow up in the SP-AQ arm were attributed to the intensity of AEs in this arm. Some cases of abdominal pain were observed in the control arm, suggesting that albendazole causes this AE. In 2016, N’diaye et al [16] reported the predominance of abdominal pain and vomiting related to SP-AQ during SMC in children aged 3 months–10 years in Senegal, and the frequencies of these AEs decreased from SMC round 1 to the subsequent rounds. Dizziness, nausea, and vomiting are the most frequent side effects, as revealed by the meta-analysis of trials on SMC using different drugs [7]. The lower frequencies of these symptoms in the DHA-PQ than in the SP-AQ arm support the previously published data on tolerance to DHA-PQ in the literature. Tolerance to DHA-PQ during SMC and clinical malaria treatment has been documented in several studies [5, 11, 17–19].

Dihydroartemisinin-PQ is well tolerated by both younger and older children (Supplementary Material 3). In the perspective of SMC extension to older children as recommended by recent WHO guidelines, DHA-PQ presents the advantages of clinical safety and tolerability among older children compared with SP-AQ. Furthermore, DHA-PQ may be more suitable than SP-AQ for mass drug administration in female adolescents aged ≥13 years owing to the risk of teratogenesis associated with the use of the latter during pregnancy. Four cases of abnormal QTc interval were recorded in each treatment arm as well as in the control arm after the 4 SMC rounds. The use of DHA-PQ has been linked to prolonged QTc interval. However, our results did not suggest a significant association between DHA-PQ and abnormal QTc, as described by several authors [5, 11, 17–20]. We assessed the hepatotoxicity of the study drugs after 3 SMC rounds by dosing the ALT level in all arms. The mean ALT was higher in the DHA-PQ than in the SP-AQ and control arms but remained within normal ranges. These data support the safety of DHA-PQ in repetitive administration, such as for SMC. The control arm exhibited higher hemoglobin levels than the SAP-AQ and DHA-PQ arms. The findings of this study are contrary to those of Zongo et al [10] who reported that the hemoglobin levels were similar in the DHA-PQ, SP-AQ, and control arms at the end of the transmission season of malaria. Furthermore, our results are inconsistent with those of Cisse et al [21] who documented the predominance of anemia in the DHA-PQ arm after SMC. Our findings highlight the role of SMC in preventing anemia in children.

The analysis performed at 4 months and at 12 months of follow up revealed no significant difference between SP-AQ and DHA-PQ in terms of efficacy against clinical malaria episodes. Both were found to be effective; however, comparing DHA-PQ and SP-AQ arms with the control arm for efficacy against clinical malaria episodes, the difference was statistically significant. Compared with the control arm, the prevalence of parasite carriage decreased after SMC round 1 and remained low for 1 month after round 4 in the DH-PQ and SP-AQ arms. A meta-analysis of 13 trials using different drugs for SMC revealed that the drugs reduced the risk of malaria infection by at least 72% and the risk of clinical malaria by 60% [7, 12, 13]. Our results for efficacy in terms of reducing the risks of clinical malaria and infection were comparable to those presented in the above studies. One of the major challenges in malaria elimination strategies is the reduction of malaria transmission via SMC. Both SP-AQ and DHA-PQ were effective in reducing the prevalence of gametocytes, which remained lower in the SP-AQ and DHA-PQ arms than in the control arm throughout the study period. These results suggest that SMC using these drugs reduces malaria transmission. Our results support that DHA-PQ is a potentially viable drug for SMC in school-aged children and that school-based SMC program is effective in reducing the burden of malaria and eliminating the disease.

### Trial Limitations

This was a randomized, clinical trial conducted with school-aged children and does not include data from younger children. Some confounding factors such as the use of bed net, the geographic location of houses, as well as the entomologic parameters were not analyzed as covariates. All drugs were administrated under the supervision of dedicated pharmacists and do not reflect the observance to treatment for larger trials with nonsupervised treatment.

## CONCLUSIONS

Data from this trial provide strong basic data to support the safety and efficacy of repetitive monthly doses of DHA-PQ in SMC and the importance to extend SMC to older children. However, these data are from one endemic area, and cluster randomized multicentric trials are needed in different endemic areas for generalizability of the results. The QTc interval was assessed before enrollment of each participant. For the mass administration of DHA-PQ in SMC, the issue related to the QTc needs further assessment.

## Supplementary Data

Supplementary materials are available at *The Journal of Infectious Diseases* online. Consisting of data provided by the authors to benefit the reader, the posted materials are not copyedited and are the sole responsibility of the authors, so questions or comments should be addressed to the corresponding author.

## Supplementary Material

jiad387_Supplementary_Data

## Notes

***Acknowledgments.*** We thank the participants and the people of Bandiagara for supporting the trial. We also thank the Bandiagara Malaria Project team and the members of the Data Safety Monitoring Board for their expertise: S Picot (the chair), JL N’diaye, MS Sissoko, Dr. A Tiono, and Dr. AH Beavogui. The results are reported according to the CONSORT guidelines (Supplementary Material 4). This work is part of the European and Developing Countries Clinical Trials Partnership EDCTP2 programme supported by the European Union (TMA2018SF-2471-RCB-DHAPQ-SMC).

***Author contributions.*** KT, DC, AKK, and MAT designed the trial, conducted the analysis, and wrote the manuscript. ST, BG, and KK performed the trial and collected the data. MD conducted the analysis and reviewed the manuscript.

***Financial support*.** Funding to pay the Open Access publication charges for this article was provided by EDCTP2 programme supported by the European Union.
