# Supplementary material for: Randomized Field Trial to Assess the Safety and Efficacy of Dihydroartemisinin-Piperaquine for Seasonal Malaria Chemoprevention in School-Aged Children in Bandiagara, Mali
Source: J Infect Dis. 2023 Sep 8;229(1):189–97. doi: 10.1093/infdis/jiad387 (PMC10786242; doi:10.1093/infdis/jiad387)
Supplement: jiad387_Supplementary_Data [file jiad387_supplementary_data.zip › Supplemental_Material-1.docx]

**Supplemental table 1 : Cumulative Number and percentage of participants experiencing Solicited events by symptom, severity and treatment arm, Intention to Treat, regardless of relationship to treatment.**

|  | | Study arms | | |
| --- | --- | --- | --- | --- |
| Symptoms | Severity | SP-AQ | DHA-PQ | Control: Albendazole |
| Fever | Mid | 1 | 4 | 1 |
|  | Moderate | 0 | 2 | 1 |
|  | Severe | 0 | 0 | 0 |
| Nausea | Mid | 9 | 6 | 0 |
|  | Moderate | 1 | 0 | 0 |
|  | Severe | 0 | 0 | 0 |
| Vomiting | Mid | 68 | 23 | 7 |
|  | Moderate | 0 | 0 | 0 |
|  | Severe | 0 | 0 | 0 |
| Headache | Mid | 87 | 50 | 27 |
|  | Moderate | 1 | 1 | 0 |
|  | Severe | 0 | 0 | 0 |
| Dizziness | Mid | 9 | 6 | 1 |
|  | Moderate | 0 | 0 | 0 |
|  | Severe | 0 | 0 | 0 |
| Myalgia | Mid | 6 | 0 | 2 |
|  | Moderate | 2 | 0 | 0 |
|  | Severe | 0 | 0 | 0 |
| Abdominal pain | Mid | 123 | 52 | 34 |
|  | Moderate | 11 | 2 | 1 |
|  | Severe | 0 | 0 | 0 |
| Anorexia | Mid | 4 | 3 | 0 |
|  | Moderate | 0 | 0 | 0 |
|  | Severe | 0 | 0 | 0 |
| Diarrhea | Mid | 1 | 3 | 1 |
|  | Moderate | 0 | 0 | 0 |
|  | Severe | 0 | 0 | 0 |

SP-AQ= Sulfadoxine-Pyriméthamine/Amodiaquine, DHA-PQ= Dihydroartemisin/Piperaquine
