# Supplementary material for: Randomized Field Trial to Assess the Safety and Efficacy of Dihydroartemisinin-Piperaquine for Seasonal Malaria Chemoprevention in School-Aged Children in Bandiagara, Mali
Source: J Infect Dis. 2023 Sep 8;229(1):189–97. doi: 10.1093/infdis/jiad387 (PMC10786242; doi:10.1093/infdis/jiad387)
Supplement: jiad387_Supplementary_Data [file jiad387_supplementary_data.zip › Supplemental_Material-2.docx]

**Supplemental table 2: Cumulative Number of participants experiencing Solicited events by symptom, severity, relationship and treatment arm, Intention to Treat.**

| **Study arms** | | | | | | | | | | | | | |
| --- | --- | --- | --- | --- | --- | --- | --- | --- | --- | --- | --- | --- | --- |
| Symptoms | Severity | SP-AQ | | | | DHA-PQ | | | | Control: Albendazole | | | |
|  |  | *Def.* | *Pro.* | *Pos.* | *Unr.* | *Def.* | *Pro.* | *Pos.* | *Unr.* | *Def.* | *Pro.* | *Pos.* | *Unr.* |
| Fever | Mid | 0 | 0 | 1 | 0 | 1 | 3 | 0 | 0 | 1 | 0 | 0 | 0 |
|  | Moderate | 0 | 0 | 0 | 0 | 1 | 1 | 0 | 0 | 0 | 0 | 1 | 0 |
|  | Severe | 0 | 0 | 0 | 0 | 0 | 0 | 0 | 0 | 0 | 0 | 0 | 0 |
| Nausea | Mid | 6 | 2 | 1 | 0 | 4 | 2 | 0 | 0 | 0 | 0 | 0 | 0 |
|  | Moderate | 0 | 1 | 0 | 0 | 0 | 0 | 0 | 0 | 0 | 0 | 0 | 0 |
|  | Severe | 0 | 0 | 0 | 0 | 0 | 0 | 0 | 0 | 0 | 0 | 0 | 0 |
| Vomiting | Mid | 46 | 22 | 1 | 0 | 15 | 8 | 1 | 2 | 3 | 4 | 0 | 1 |
|  | Moderate | 0 | 1 | 0 | 0 | 0 | 0 | 0 | 0 | 0 | 0 | 0 | 0 |
|  | Severe | 0 | 0 | 0 | 0 | 0 | 0 | 0 | 0 | 0 | 0 | 0 | 0 |
| Headache | Mid | 26 | 53 | 2 | 6 | 11 | 22 | 11 | 6 | 9 | 10 | 1 | 7 |
|  | Moderate | 1 | 0 | 0 | 0 | 0 | 1 | 0 | 0 | 0 | 0 | 0 | 0 |
|  | Severe | 0 | 0 | 0 | 0 | 0 | 0 | 0 | 0 | 0 | 0 | 0 | 0 |
| Dizziness | Mid | 2 | 7 | 0 | 0 | 1 | 5 | 0 | 0 | 1 | 0 | 0 | 0 |
|  | Moderate | 0 | 0 | 0 | 0 | 0 | 0 | 0 | 0 | 0 | 0 | 0 | 0 |
|  | Severe | 0 | 0 | 0 | 0 | 0 | 0 | 0 | 0 | 0 | 0 | 0 | 0 |
| Myalgia | Mid | 3 | 3 | 0 | 0 | 0 | 0 | 0 | 0 | 2 | 1 | 0 | 0 |
|  | Moderate | 0 | 2 | 0 | 0 | 0 | 0 | 0 | 0 | 0 | 0 | 0 | 0 |
|  | Severe | 0 | 0 | 0 | 0 | 0 | 0 | 0 | 0 | 0 | 0 | 0 | 0 |
| Abdominal pain | Mid | 58 | 60 | 3 | 3 | 20 | 24 | 3 | 5 | 11 | 15 | 1 | 6 |
|  | Moderate | 3 | 7 | 0 | 0 | 1 | 1 | 0 | 0 | 0 | 1 | 1 | 0 |
|  | Severe | 0 | 0 | 0 | 0 | 0 | 0 | 0 | 0 | 0 | 0 | 0 | 0 |
| Anorexia | Mid | 2 | 2 | 0 | 0 | 0 | 3 | 0 | 0 | 0 | 0 | 0 | 0 |
|  | Moderate | 0 | 0 | 0 | 0 | 0 | 0 | 0 | 0 | 0 | 0 | 0 | 0 |
|  | Severe | 0 | 0 | 0 | 0 | 0 | 0 | 0 | 0 | 0 | 0 | 0 | 0 |
| Diarrhea | Mid | 1 | 0 | 0 | 0 | 0 | 3 | 0 | 0 | 0 | 1 | 0 | 0 |
|  | Moderate | 0 | 0 | 0 | 0 | 0 | 0 | 0 | 0 | 0 | 0 | 0 | 0 |
|  | Severe | 0 | 0 | 0 | 0 | 0 | 0 | 0 | 0 | 0 | 0 | 0 | 0 |

*Unr: Unrelated, Pos: Possible, Pro: Probable, Def: Definite, Rel: Related as possible+probable+definite*
