# Supplementary material for: Randomized Field Trial to Assess the Safety and Efficacy of Dihydroartemisinin-Piperaquine for Seasonal Malaria Chemoprevention in School-Aged Children in Bandiagara, Mali
Source: J Infect Dis. 2023 Sep 8;229(1):189–97. doi: 10.1093/infdis/jiad387 (PMC10786242; doi:10.1093/infdis/jiad387)
Supplement: jiad387_Supplementary_Data [file jiad387_supplementary_data.zip › Supplemental_Material-3.docx]

**Supplemental table 3: cumulative events by age groups and by treatment arm throughout the 4 SMC rounds**

| Drug regiment | Age group in Years | Solicited events | | | | Total events |
| --- | --- | --- | --- | --- | --- | --- |
|  |  | Abdominal pain | Headaches | Vomiting | Nausea |  |
| SP-AQ | 6-10 (n=65) | 66 | 28 | 23 | 1 | 118 |
|  | 11-15 (n=51) | 68 | 59 | 47 | 9 | 183 |
| DHA-PQ | 6-10 (n=66) | 35 | 28 | 14 | 3 | 80 |
|  | 11-15 (n=48) | 19 | 23 | 12 | 3 | 57 |
| Control (AL) | 6-10 (n=68) | 26 | 17 | 4 | 0 | 47 |
|  | 11-15 (n=47) | 9 | 10 | 2 | 0 | 21 |
